# Supplementary material for: Transcriptome Analysis Reveals Vimentin-Induced Disruption of Cell–Cell Associations Augments Breast Cancer Cell Migration
Source: Cells. 2022 Dec 13;11(24):4035. doi: 10.3390/cells11244035 (PMC9776984; doi:10.3390/cells11244035)
Supplement: Supplementary file 1 [file cells-11-04035-s001.zip › DEGs and enrichment analysis.zip!/DEGs and enrichment analysis/WT_VIMvsVector KEGG/GSEA/WT_VIM/RNA_POLYMERASE/RNA_POLYMERASE(HSA03020).html]

Details for gene set RNA\_POLYMERASE(HSA03020)[GSEA]

|  || Dataset | fpkm.sample |
| Phenotype | sample.cls |
| Upregulated in class | WT\_VIM |
| GeneSet | RNA\_POLYMERASE(HSA03020) |
| Enrichment Score (ES) | 0.68084145 |
| Normalized Enrichment Score (NES) | 1.202488 |
| Nominal p-value | 0.0 |
| FDR q-value | 0.2499995 |
| FWER p-Value | 0.335 |
Table: GSEA Results Summary

  

Fig 1: Enrichment plot: RNA\_POLYMERASE(HSA03020)      
 Profile of the Running ES Score & Positions of GeneSet Members on the Rank Ordered List

  

| PROBE | DESCRIPTION (from dataset) | GENE SYMBOL | GENE\_TITLE | RANK IN GENE LIST | RANK METRIC SCORE | RUNNING ES | CORE ENRICHMENT || 1 | ENSG00000105258 | POLR2I |  |  | 3191 | 4.304 | 0.0215 | Yes |
| 2 | ENSG00000177700 | POLR2L |  |  | 3790 | 3.286 | 0.0690 | Yes |
| 3 | ENSG00000058600 | POLR3E |  |  | 4245 | 2.825 | 0.1108 | Yes |
| 4 | ENSG00000161980 | POLR3K |  |  | 4290 | 2.789 | 0.1590 | Yes |
| 5 | ENSG00000099817 | POLR2E |  |  | 4392 | 2.736 | 0.2052 | Yes |
| 6 | ENSG00000100142 | POLR2F |  |  | 4690 | 2.564 | 0.2451 | Yes |
| 7 | ENSG00000181222 | POLR2A |  |  | 5399 | 2.234 | 0.2723 | Yes |
| 8 | ENSG00000100413 | POLR3H |  |  | 5782 | 2.104 | 0.3027 | Yes |
| 9 | ENSG00000168002 | POLR2G |  |  | 6127 | 1.997 | 0.3319 | Yes |
| 10 | ENSG00000257545 | AC079385.1 |  |  | 6356 | 1.959 | 0.3624 | Yes |
| 11 | ENSG00000271754 | AL355802.2 |  |  | 6559 | 1.909 | 0.3925 | Yes |
| 12 | ENSG00000137054 | POLR1E |  |  | 6681 | 1.882 | 0.4234 | Yes |
| 13 | ENSG00000005075 | POLR2J |  |  | 7106 | 1.791 | 0.4477 | Yes |
| 14 | ENSG00000171453 | POLR1C |  |  | 8363 | 1.567 | 0.4539 | Yes |
| 15 | ENSG00000102978 | POLR2C |  |  | 8477 | 1.550 | 0.4791 | Yes |
| 16 | novel.232 | - |  |  | 9320 | 1.440 | 0.4901 | Yes |
| 17 | ENSG00000272655 | POLR2J4 |  |  | 9939 | 1.366 | 0.5036 | Yes |
| 18 | novel.324 | - |  |  | 10127 | 1.343 | 0.5240 | Yes |
| 19 | ENSG00000068654 | POLR1A |  |  | 10855 | 1.279 | 0.5341 | Yes |
| 20 | ENSG00000125630 | POLR1B |  |  | 11277 | 1.239 | 0.5487 | Yes |
| 21 | ENSG00000168495 | POLR3D |  |  | 11922 | 1.188 | 0.5586 | Yes |
| 22 | ENSG00000186141 | POLR3C |  |  | 12055 | 1.177 | 0.5771 | Yes |
| 23 | ENSG00000163882 | POLR2H |  |  | 12121 | 1.172 | 0.5965 | Yes |
| 24 | ENSG00000113356 | POLR3G |  |  | 12356 | 1.152 | 0.6127 | Yes |
| 25 | ENSG00000144231 | POLR2D |  |  | 12500 | 1.141 | 0.6303 | Yes |
| 26 | ENSG00000147669 | POLR2K |  |  | 13393 | 1.073 | 0.6340 | Yes |
| 27 | ENSG00000121851 | POLR3GL |  |  | 13897 | 1.034 | 0.6436 | Yes |
| 28 | ENSG00000148606 | POLR3A |  |  | 14129 | 1.016 | 0.6575 | Yes |
| 29 | ENSG00000132664 | POLR3F |  |  | 16396 | 0.894 | 0.6349 | Yes |
| 30 | ENSG00000013503 | POLR3B |  |  | 16466 | 0.890 | 0.6493 | Yes |
| 31 | ENSG00000047315 | POLR2B |  |  | 16916 | 0.862 | 0.6568 | Yes |
| 32 | ENSG00000105849 | TWISTNB |  |  | 16990 | 0.858 | 0.6706 | Yes |
| 33 | ENSG00000228049 | POLR2J2 |  |  | 17258 | 0.841 | 0.6808 | Yes |
| 34 | ENSG00000258284 | POLR2KP1 |  |  | 19386 | 0.721 | 0.6575 | No |
| 35 | ENSG00000285437 | POLR2J3 |  |  | 20256 | 0.673 | 0.6546 | No |
| 36 | ENSG00000228600 | POLR2CP1 |  |  | 28925 | 0.000 | 0.5079 | No |
| 37 | ENSG00000066379 | ZNRD1 |  |  | 32072 | NaN | 0.4548 | No |
| 38 | ENSG00000280778 | AC243547.3 |  |  | 32086 | NaN | 0.4547 | No |
| 39 | ENSG00000279504 | AD001527.2 |  |  | 35044 | NaN | 0.4049 | No |
| 40 | ENSG00000232090 | AC016724.1 |  |  | 36496 | NaN | 0.3805 | No |
| 41 | ENSG00000274446 | AP001005.2 |  |  | 36529 | NaN | 0.3801 | No |
| 42 | ENSG00000218313 | AL139274.1 |  |  | 37131 | NaN | 0.3701 | No |
| 43 | ENSG00000255210 | AP001889.1 |  |  | 37152 | NaN | 0.3700 | No |
| 44 | ENSG00000265469 | POLR3KP2 |  |  | 37582 | NaN | 0.3629 | No |
| 45 | ENSG00000224510 | POLR2KP2 |  |  | 39548 | NaN | 0.3298 | No |
| 46 | ENSG00000244565 | AC073901.1 |  |  | 39776 | NaN | 0.3261 | No |
| 47 | ENSG00000276388 | AL591424.2 |  |  | 40532 | NaN | 0.3135 | No |
| 48 | ENSG00000268295 | POLR3GP1 |  |  | 40939 | NaN | 0.3068 | No |
| 49 | ENSG00000271315 | AC174048.1 |  |  | 48968 | NaN | 0.1711 | No |
| 50 | novel.236 | - |  |  | 48980 | NaN | 0.1711 | No |
| 51 | ENSG00000214626 | POLR3DP1 |  |  | 50532 | NaN | 0.1450 | No |
| 52 | ENSG00000266984 | POLR3GP2 |  |  | 52941 | NaN | 0.1045 | No |
| 53 | ENSG00000238211 | POLR2LP1 |  |  | 57316 | NaN | 0.0306 | No |
| 54 | ENSG00000232647 | POLR3KP1 |  |  | 59069 | NaN | 0.0011 | No |
Table: GSEA details [plain text format]

  

Fig 2: RNA\_POLYMERASE(HSA03020)      
 Blue-Pink O' Gram in the Space of the Analyzed GeneSet

  

Fig 3: RNA\_POLYMERASE(HSA03020): Random ES distribution      
 Gene set null distribution of ES for **RNA\_POLYMERASE(HSA03020)**
